# Kv2 channels create endoplasmic reticulum / plasma membrane junctions: a brief history of Kv2 channel subcellular localization

**DOI:** 10.1080/19336950.2019.1568824

**Published:** 2019-02-02

**Authors:** Ben Johnson, Ashley N. Leek, Michael M. Tamkun

**Affiliations:** a Molecular, Cellular and Integrative Neurosciences Graduate Program, Colorado State University, Fort Collins, CO, USA; b Department of Biomedical Sciences, Colorado State University, Fort Collins, CO, USA; c Department of Biochemistry and Molecular Biology, Colorado State University, Fort Collins, CO, USA

**Keywords:** Kv2.1, Kv2.2, ER/PM junction, VAP, membrane contact site

## Abstract

The potassium channels Kv2.1 and Kv2.2 are widely expressed throughout the mammalian brain. Kv2.1 provides the majority of delayed rectifying current in rat hippocampus while both channels are differentially expressed in cortex. Particularly unusual is their neuronal surface localization pattern: while half the channel population is freely-diffusive on the plasma membrane as expected from the generalized Singer & Nicolson fluid mosaic model, the other half localizes into micron-sized clusters on the soma, dendrites, and axon initial segment. These clusters contain hundreds of channels, which for Kv2.1, are largely non-conducting. Competing theories of the mechanism underlying Kv2.1 clustering have included static tethering to being corralled by an actin fence. Now, recent work has demonstrated channel clustering is due to formation of endoplasmic reticulum/plasma membrane (ER/PM) junctions through interaction with ER-resident VAMP-associated proteins (VAPs). Interaction between surface Kv2 channels and ER VAPs groups channels together in clusters. ER/PM junctions play important roles in inter-organelle communication: they regulate ion flux, are involved in lipid transfer, and are sites of endo- and exocytosis. Kv2-induced ER/PM junctions are regulated through phosphorylation of the channel C-terminus which in turn regulates VAP binding, providing a rapid means to create or dismantle these microdomains. In addition, insults such as hypoxia or ischemia disrupt this interaction resulting in ER/PM junction disassembly. Kv2 channels are the only known plasma membrane protein to form regulated, injury sensitive junctions in this manner. Furthermore, it is likely that concentrated VAPs at these microdomains sequester additional interactors whose functions are not yet fully understood.

“[I]n order to achieve a satisfactory understanding of how any biological system functions, the detailed molecular composition and structure of that system must be known”

-Singer & Nicolson, 1972 [1]

## Introduction

Kv2.1 was first discovered by cDNA expression cloning from rat brain in Rolf Joho’s lab in 1989 [2]. This channel, originally designated drk1, encoded a classic delayed rectifier K^+^ channel with a high activation threshold of −15 mV. These investigators postulated that the unusually large cytoplasmic C-terminus may be involved in both channel regulation and subcellular localization. As discussed below, these ideas were correct. Three years later in the Snyder lab, Kv2.2, first termed cdrk1, was cloned from rat taste buds using drk1 sequence to screen a cDNA library [3]. Kv2.1 and Kv2.2 are conserved in the channel forming core, containing the six transmembrane domains and ion conducting pore, and within a limited region of the proximal C-terminus, but otherwise show little sequence identity [3]. mRNA expression analysis indicated areas of overlapping expression in the CNS but suggested that cells predominantly use either Kv2.1 or Kv2.2 [4,5]. Studies of Kv2.1 have dominated the Kv2 literature, partially due to a Kv2.2 cloning artifact which wasn’t realized for some time [6], and Kv2.1 is emphasized in the discussion presented below. However, Kv2.2 shares many properties with Kv2.1, as well as some intriguing differences, which are noted where appropriate.

RNA expression studies and antibody-based protein detection indicate that Kv2 channels are perhaps the most widely expressed voltage-gated K^+^ channel genes in terms of tissue distribution. They are present in cortical [7], hippocampal [8], and α-motoneurons [9] but also in retinal bipolar cells [10], cardiac myocytes [11], vascular and gastrointestinal smooth muscle [12,13], and pancreatic beta cells [14,15]. Kv2.1 protein expression is high not only in neurons but even in cell types such as vascular smooth muscle where the outward currents average 100–200 pA [12]. From a functional standpoint, Kv2.1-derived K^+^ currents regulate the action potential waveform in pyramidal neurons undergoing high frequency stimulation [16], cardiac action potential duration [17], vascular smooth muscle membrane potential and contraction [12], beta cell potential and insulin release [14,15,18], and cortical neuron apoptosis [19–22]. Mutations altering Kv2.1 conductance are linked to epilepsy in humans [23,24] and Kv2.1 knockout mice are epileptic, hyperactive, and display defects in spatial learning [25]. Additionally, these mice have elevated serum insulin levels, a prolonged glucose-induced beta cell action potential duration and a diminished firing frequency [18]. However, this review will not focus on the electrical activity of Kv2 channels but will emphasize the non-conducting and structural roles of these channels to induce stable, micron-sized endoplasmic reticulum/plasma membrane junctions that likely function as sites for specialized Ca^2+^ homeostasis and membrane trafficking.

## Kv2 channels “cluster” on the membrane

Two years after the cloning of Kv2.1 cDNA the Trimmer group used immunolocalization in rat cerebral cortex to explore the distribution of Kv2.1 [26]. Immunoreactivity was described as neuronal specific, with staining present on the soma and both the apical and proximal dendrites. Importantly, Kv2.1 immuno-staining was described as having a “punctate, membrane-associated nature” as opposed to a fully diffuse localization across somato-dendritic compartments. This was the first evidence that Kv2.1 adopts a unique and enigmatic subcellular localization pattern on the neuronal plasma membrane. Two years later these findings were both supported and expanded upon by the Snyder group that first identified Kv2.2 [4,5]. Whereas the Trimmer group initially focused on pyramidal cells of the rat cortex, Hwang et al. also found α-Kv2.1 staining in Purkinje and granule cells of the cerebellum and granule cells of the olfactory bulb. Furthermore, the Snyder group reported molecular layer staining consistent with stellate and basket cells. Again, Kv2.1 immunoreactivity was described as “punctate” in nature. Given the additional finding that Kv2.1 migrates heterogeneously during SDS gel electrophoresis, the Snyder lab postulated that differences in cellular localization could be due to either Kv2.1 posttranslational modification or “may result from some intrinsic targeting information contained within the amino acid sequences of the two K^+^ channels that directs them to the different subcellular compartments where they are then post-translationally modified” [5]. These hypotheses would prove especially apt in the years to come.

In 1996, evidence suggesting a specific domain of Kv2.1 is important for its localization, as well as the putative role of posttranslational modification, began to solidify [27]. The Trimmer group generated two Kv2.1 C-terminal truncation mutants, ∆C187 and ∆C318, and found that while wildtype Kv2.1 and the ∆C187 mutant both formed 0.5–1.0 µm “clusters” on the cell membrane of MDCK cells, removal of additional amino acids from the C-terminus abolished clustering, as the ∆C318 construct was uniformly distributed. It was also revealed that while wildtype Kv2.1 would cluster in MDCK cells, the clustered phenotype was reported absent in COS-1 cells. Concomitantly, Scannevin et al. noted differences in the gel mobility of Kv2.1 in the two separate cell types, i.e. Western analysis revealed a higher molecular weight in the clustering MDCK cells compared to a lower molecular weight in the non-clustering COS-1 cells, consistent with a link between channel posttranslational modification and clustering. Given earlier findings using *in vivo* ^32^P-labeling [28], it was deemed likely a portion of these posttranslational modifications represented phosphorylation events.

At this time, the mechanism by which Kv2.1 localized into clusters was poorly understood, for all that was known was that a portion of the C-terminus was critical and that clustering may be phosphorylation-dependent. Then in 2000, the Trimmer lab used a series of truncation and internal deletion mutations to identify a 26 amino acid region within the C-terminus of Kv2.1 that was responsible for clustering [29]. They called this 26 amino acid region the proximal restriction and clustering (PRC) domain. The addition of this domain to the C-terminus of Kv1.5 endowed a clustering phenotype to its otherwise fully diffuse membrane localization while its removal completely abolished clustering in Kv2.1. In addition, several point mutations within the PRC domain also abrogated the clustering phenotype.

Kv2.2 also contains a PRC-like domain and clusters on the membranes of neurons, however it would take 18 years from the time it was first cloned for the field to recognize this fact due to an unfortunate single-nucleotide deletion that resulted in a diffuse truncated protein [6].

As it was previously established that Kv2.1 channels are capable of shaping dendritic [Ca^2+^]_i_ transients [8], Antonucci et al. in 2001 [30], examined whether Kv2.1 colocalized with Ca^2+^ signaling proteins. They reported colocalization between Kv2.1 clusters and both calsequestrin and ryanodine receptors in pyramidal neurons. In addition, overexpression of a GFP-Kv2.1 construct via adenovirus altered the localization of both calsequestrin and ryanodine receptors, causing them to more closely resemble the exact distribution of Kv2.1 macroclusters. This effect of Kv2.1 overexpression suggested a direct or indirect association between Kv2.1 and these two Ca^2+^ signaling proteins.

## Kv2 clustering is regulated by neuronal activity and insult

In 2004, Misonou et al. [31], proposed a link between neuronal electrical activity, Kv2.1 phosphorylation, and somatic clustering. First, they observed that the induction of class 5 motor seizures by kainate injection declustered Kv2.1 *in vivo*. Next, 10 µM glutamate treatment of cultured hippocampal neurons to increase spontaneous bursting activity caused a similar declustering with no effect on the total surface level of Kv2.1 protein. These two findings effectively coupled the electrical activity of the cell with the subcellular localization of Kv2.1. The glutamate-induced declustering was also associated with a coincident decrease in the phosphorylation state of the channel; both dephosphorylation and declustering were dosage- and time-dependent and reversible with glutamate washout. Pre-incubation with 100 µM AP-5 (D-2-amino-5-phosphonopentanoate) and 10 µM CNQX (6-cyano-7-nitroquinoxaline-2,3-dione) prior to glutamate treatment blocked dephosphorylation and declustering, suggesting that glutamate was signaling these downstream events through NMDA and AMPA-kainate glutamate receptors. In addition, removal of Ca^2+^ from the media or Ca^2+^ channel block with CdCl_2_ blunted glutamate-induced dephosphorylation and declustering.

**Figure 1.**
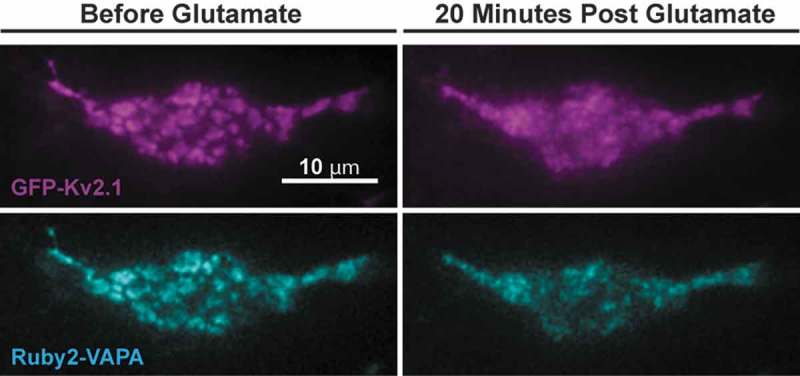
Glutamate-induced Kv2.1 and VAP declustering in cultured hippocampal neurons. The effect of 20 μM glutamate on GFP-Kv2.1 and Ruby2-VAPA co-localization on the basal membrane of transfected DIV 8 rat hippocampal neurons is illustrated. Cells were transfected with GFP-Kv2.1 and Ruby2-VAPA and after 24 hrs imaged via TIRF microscopy. The top row illustrates the glutamate dephosphorylation-induced Kv2.1 declustering while the bottom row illustrates the VAP unbinding that occurs in parallel.

Misonou et al. next identified the protein phosphatases responsible for Kv2.1 dephosphorylation. Treatment with okadaic acid, an inhibitor of protein phosphatase 1 and protein phosphatase 2A increased phosphorylation in unstimulated cultures, but failed to prevent glutamate-induced dephosphorylation of the channel. Cyclosporin A, on the other hand, an inhibitor of calcineurin, had no effect on phosphorylation in unstimulated cultures, but did abolish glutamate-induced dephosphorylation and declustering. This led to the conclusion that “glutamate-induced dephosphorylation of Kv2.1 and lateral translocation of Kv2.1 from a clustered to uniform localization are tightly coupled and occur by means of ionotropic glutamate receptor stimulation, leading to Ca^2+^-dependent activation of calcineurin.” In two 2005 papers [32,33], Misonou and coworkers discovered that Kv2.1 cluster dispersal also occurred after hypoxic or ischemic insult. They postulated that “Kv2.1 clusters form specialized ‘micro-signaling domains’ to regulate somatodendritic Ca^2+^ signaling at these sites” [32]. The glutamate-induced declustering of Kv2.1 is illustrated in the top row of Figure 1.

Contrary to Kv2.1, Kv2.2 is largely resistant to insult-induced declustering. In both mouse brain sections and HEK293 cells, Kv2.2 clusters largely remain intact after hypoxia and elevated cytoplasmic Ca^2+^ [7]. This resistance to declustering is paired with less phosphorylation at rest, as well as less dephosphorylation during insult [7]. The significance of this difference in sensitivity to declustering stimuli, if one exists, has yet to be examined.

## Relationship between Kv2.1 clustering and channel activity

Throughout early studies of Kv2.1 localization there were few solid ideas with respect to the physiological significance of Kv2.1 clustering. However, glutamate treatment affected more than just the phosphorylation state and subcellular localization of Kv2.1. In the final finding of the Misonou et al., 2004 paper, they concluded that glutamate-induced declustering resulted in a > 20 mV hyperpolarizing shift in the V_1/2_ of neuronal I_K_, from +15.5 ± 0.5 mV to −8.4 ± 0.5 mV. This shift was reversible after a 2 h glutamate washout and was blocked by calcineurin inhibitors. This last conclusion shaped the field’s thinking in regards to the physiological significance of Kv2.1 clustering, effectively linking the channel’s voltage-sensitivity to its subcellular localization. The next year Misonou et al. went further, hypothesizing that clusters on the soma and proximal dendrites acted as “on/off” switches that regulated the intrinsic excitability of the cell [33]. It was thought at the time that clusters represented reservoirs of high-threshold Kv2.1 channels, ready to be hyper-activated upon cluster release when needed. Throughout the early 2000s, the overall hypothesis was that Kv2.1 was tethered to cytoskeletal elements adjacent to the ER and near ionotropic receptors, kinases, and phosphatases that served to either maintain or disrupt Kv2.1 clustering and thus regulate channel voltage-dependence [34]. As is often the case, a more complex picture arose as we learned more.

Working under the assumption that Kv2.1 clustering regulated the voltage-dependence of channel activation, our group, the Tamkun laboratory, began cell biological studies of Kv2.1 behavior on the membrane surface to better understand the mechanism and dynamics underlying Kv2.1 localization and the regulation of channel function. Surprisingly, both traditional FRAP approaches and newly developed single molecule, quantum dot-based, imaging indicated that Kv2.1 channels were not statically tethered within clusters at the cell surface as originally proposed [34], but rather were corralled within a cytoskeletal fence [35]. Here the clustered channels had a lateral mobility similar to Kv2.1 outside the clusters, which agreed with that expected for freely diffusing membrane proteins. Thus, it seemed likely that phosphorylation of the C-terminus allowed Kv2.1 to interact with an additional unknown protein and this additional mass prohibited the channel from diffusing through the cluster’s perimeter boundary. Non-clustered channels did not have this posttranslational modification, could not interact with this unknown accessory protein, and therefore were too small to be hindered by the cytoskeletal architecture and were free to diffuse into and out of clusters. Supporting this hypothesis were the dual findings that in HEK293 cells Kv2.1 clusters tend to be entirely bordered by actin filaments and that disruption of actin with swinholide A dissolved Kv2.1 clusters [36]. A mechanism by which Kv2.1 was corralled in a phosphorylation-dependent manner by a cytoskeletal perimeter fence neatly fit all available data at the time.

Since these data cast doubt onto the prevailing model for Kv2.1 clusters, i.e. where static tethering regulates the voltage-dependence of activation, we decided to directly examine the function of clustered Kv2.1 using HEK293 cells expressing GFP-Kv2.1 and a cell-attached patch clamp approach [37]. While cell-attached patches on membrane regions devoid of Kv2.1 clusters, but populated with freely diffusing Kv2.1 channels, exhibited macroscopic delayed rectifier currents, little to no channel activity was detected when the patch clamp pipet was placed directly onto Kv2.1 clusters, as if the clustered channels were non-conducting even at +60 mV. When comparing the whole cell gating current magnitude to ionic currents in transfected HEK293 cells a striking mismatch was found, suggesting that all the Kv2.1 channels responded to a depolarizing stimulus with a moving S4 domain but only a small percentage of these transitions resulted in pore opening. This finding agreed well with early work from the Pongs lab [38] that indicated a discrepancy between Kv2.1 gating and ionic current in *Xenopus laevis* oocytes, where less than 1% of the gating channels actually opened. To test whether Kv2.1 clusters acted as reservoirs of non-conducting channels that were activated upon release, we next measured whole cell currents before and after inducing Kv2.1 declustering via either actin depolymerization to dissolve the hypothesized diffusion-limiting fence, or alkaline phosphatase in the patch clamp pipet to dephosphorylate the clustered channel [37]. Both treatments resulted in declustering, however while the alkaline phosphatase treatment resulted in the expected shift of voltage dependence, declustering via actin depolymerization did not. Neither treatment increased current density, which would be expected if non-conducting channels suddenly became conducting once declustered. These findings were contrary to the prevailing theories about the channel, as they demonstrated that clustering per se has little impact on channel function. While phosphorylation seems to both govern some aspects of channel electrical activity as well as clustering, location and conductance were not inextricably linked.

Following studies would confirm these findings. Baver and O’Connell [39] showed that the NMDA receptor-based regulation of Kv2.1 activity occurs in the absence of Kv2.1 clustering. In addition, our group would later find that the non-conducting state was regulated by surface channel density and not location on the cell surface [40]. The non-conducting state existed in C-terminal truncation mutants that lack the PRC domain and cannot cluster and the percentage of non-conducting channels increased as a function of surface channel number [40]. Further supporting a separation between localization and conductance, in 2015 the Trimmer lab found that the cell cycle-dependent regulation of Kv2.1 clustering in COS-1 cells, which is due to changes in Kv2.1 phosphorylation, does not affect Kv2.1 currents [41]. While we now know that uncoupling of S4 movement from pore opening is regulated by channel density, the exact mechanism underlying this disconnect remains a mystery.

## Non-conducting functions of Kv2.1 clusters

If the clustered channels are not, and do not become, conducting upon declustering, what is their purpose, especially considering the gating current data that indicates non-conducting Kv2.1 channels still sense changes in membrane potential? The high levels of Kv2.1 protein in multiple cell types suggest a structural role and these high levels would also mandate the non-conducting state, for without this, neurons would be electrically silenced.

Non-conducting Kv2.1 had already been linked to exocytosis, for the Lotan group found that Kv2.1 facilitates dense core vesicle release from neuroendocrine cells independently of potassium flux via Kv2.1 interaction with syntaxin [42,43]. Unfortunately, since this work did not employ imaging, no relationship was drawn between these results and Kv2.1 localization. Motivated by this Lotan work, our lab next asked whether the Kv2.1 clusters acted as insertion platforms for membrane protein delivery to the plasma membrane [44]. Approximately 85% of both Kv2.1 and Kv1.4 channel plasma membrane insertion events occurred at the Kv2.1 cluster perimeter. As Kv1.4 is freely diffuse, this localized delivery is not specific to cluster-resident proteins. In addition, since endocytosis was also observed at the perimeter of Kv2.1 clusters, these microdomains were postulated to act as membrane trafficking hubs [44,45]. Very recent work from the MacDonald and Gaisano labs [14,15] further demonstrates that Kv2.1 clusters regulate insulin exocytosis in pancreatic beta cells.

Du and colleagues [8], using a combination of immunohistochemical and electron microscopy approaches, had previously found that Kv2.1 clusters were often localized on neuronal cell membranes directly apposed to both ER/PM junctions and astrocyte membranes. These junctions, or discs of flattened cortical ER located 5–8 nm from the intracellular side of the cell membrane, were continuous with either the smooth or rough ER. This association between Kv2.1 clusters and membrane junctions had also been reported in rat α-motoneurons where the Kv2.1 clusters are apposed to cholinergic C-type synaptic terminals [9]. As discussed above, the Trimmer group had reported association between Kv2.1 clusters and ER proteins such as ryanodine receptors. Thus, still searching for non-conducting functions associated with Kv2.1 clusters, our lab returned to the idea that Kv2.1 clusters existed at sites of ER/PM junctions as first seen by Du et al. in 1998. Given that the clustered, but non-conducting, Kv2.1 channels still likely responded to voltage, and being influenced by the work of Kurt Beam [46], we wondered whether they could act in a fashion similar to L-type Ca^2+^ channels at the skeletal muscle triad junction where Ca^2+^ channels couple membrane depolarization to Ca^2+^ release from internal stores via mechanical activation of ryanodine receptors. Therefore, we further examined the relationship between Kv2.1 and the ER.

In untransfected HEK293 cells, endogenous ER/PM junctions were small in size and transient in nature as observed using TIRF imaging of fluorescent ER markers as well as electron microscopy [47]. In contrast, following Kv2.1 expression, cortical ER adjacent to the Kv2.1 clusters was dramatically remodeled. These results suggested that Kv2.1 binds to the ER surface and tethers the ER to the PM. This tethering generates the observed Kv2.1 cluster phenotype and explains the high Kv2.1 mobility within the clusters, assuming the ER binding partner, whether it be protein or lipid, is mobile within the ER membrane. Thus, Kv2.1 channel bound to the ER is corralled by the edge of the ER membrane at the point where this organelle turns inward towards the more internal cytoplasm. Further confirming a non-conducting role of Kv2.1 is to induce ER/PM junctions, glutamate-induced Kv2.1 declustering in cultured hippocampal neurons resulted in concomitant retraction of ER localized under Kv2.1 clusters after cluster dispersal as illustrated in [47]. The next obvious step required to understand the Kv2.1-ER relationship was for us to identify the Kv2.1 binding partner on the ER cytoplasmic face, be that protein or lipid. As both Kv2.1 and Kv2.2 form clusters, our lab privately hypothesized that a common mechanism behind this clustering existed and that Kv2.2 also likely interacted with an unknown ER resident to form ER/PM junctions. Indeed, recent efforts have confirmed that cortical ER remodeling is a function of both Kv2.1 and Kv2.2 [48,49].

## Kv2 channels and the VAPs

With the hope that Kv2.1 interacts with an ER protein, our group used APEX-based proximity biotinylation and identified a 33 kD protein that likely represented the Kv2.1 binding partner [48]. With critical input from Tim Levine of University College, London, and using a combination of redistribution assays, FRET, and siRNA knockdown experiments, we confirmed that this 33 kD protein was the ER VAMP-associated protein, or VAP. We also found, as expected, that both Kv2.1 and Kv2.2 interact with VAPs. It is this Kv2-VAP interaction which tethers the ER to the PM and is thus responsible for Kv2 clustering. For additional information, see Johnson et al. [48].

VAP was first discovered in *Aplysia californica* and was originally referred to as VAP-33 as the polypeptide migrated at 33 kD during SDS gel electrophoresis [50]. Four years later, three mammalian homologues were uncovered: VAPA, VAPB, and VAPC [51]. While VAPA was almost identical to the *Aplysia* VAP-33, VAPB and VAPC were novel homologues with the latter, VAPC, being a VAPB splice variant that lacks the transmembrane domain found at the C-terminus of VAP A and B [51,52]. All VAP proteins contain an immunoglobulin-like β sheet on their N-terminus followed by a coiled-coil domain [53]. A VAP consensus sequence, FK(V/I)KTT(VA)**P**(K/R)(K/R)YCV(K/R)P, within their N-terminal major sperm protein (MSP) domain may contribute to VAP oligomerization [54] in addition to the transmembrane domain [51,55]. A missense mutation within VAPB (P56S; highlighted in the above sequence) causes intracellular aggregation of the VAPB mutant and is associated with late-onset spinal muscular atrophy and amyotrophic lateral sclerosis [56]. This mutation recruits both wildtype VAPB and VAPA to these immobile aggregates and results in cell death in culture systems [57]. In addition, the P56S mutation interferes with VAPBs ability to interact with other proteins via its MSP domain [58]. Deletion of the *Drosophila* homolog, DVAP-33A, results in a decrease in the number of synaptic boutons while overexpression leads to an increase in bouton number and decrease in average size [59]. There is also a rich literature connecting the VAPs to viral replication which raises the question of whether Kv2-induced ER/PM junctions are in some way co-opted for viral transmission [60–62].

**Figure 2.**
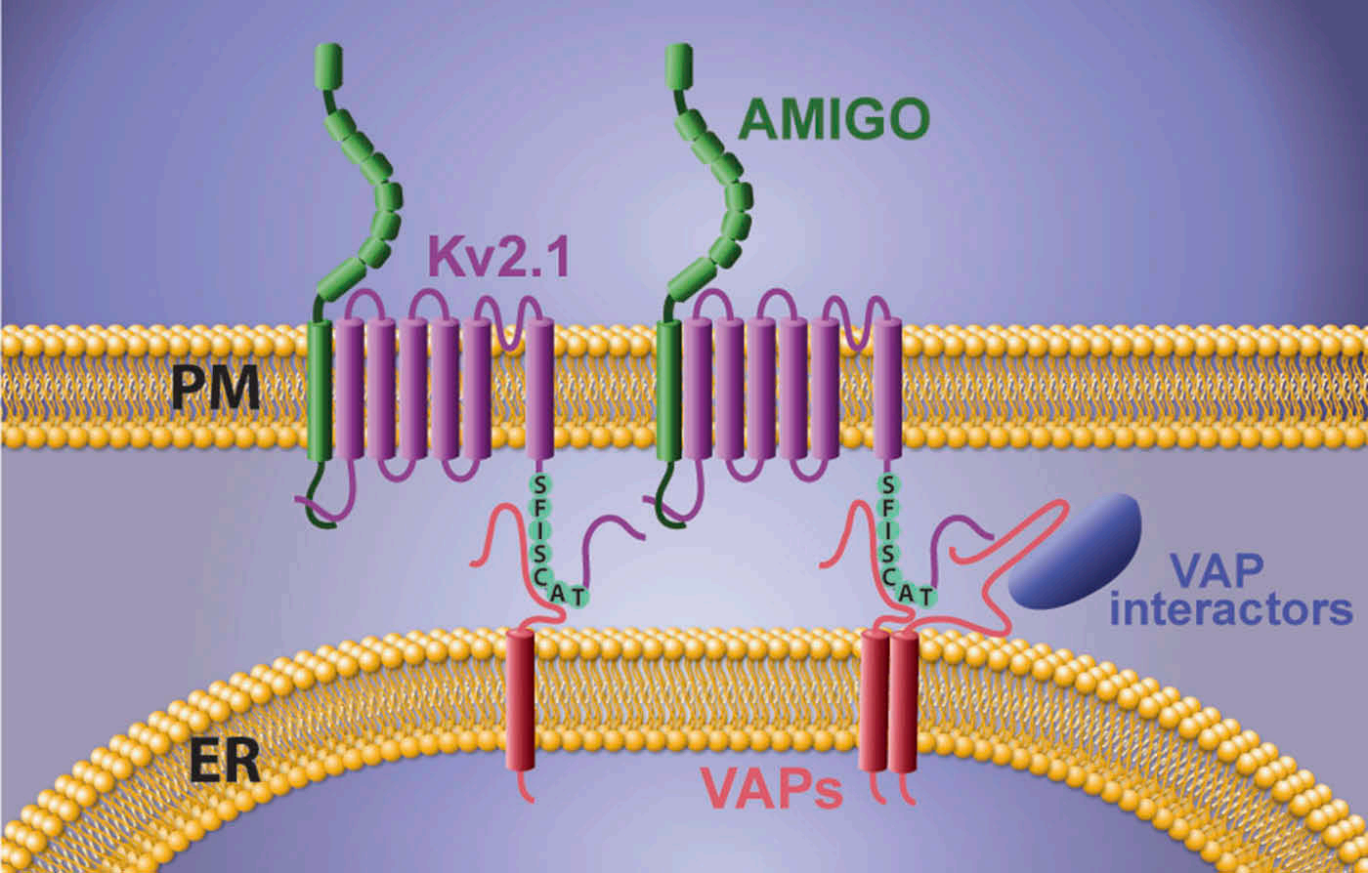
Mechanism of ER/PM junction formation and Kv2 clustering. The binding of a noncanonical FFAT motif (SFISCAT in Kv2.1, pictured here) within the Kv2 C-terminus to VAPs embedded within the cortical ER membrane generates both membrane contact site formation and the Kv2 clustered phenotype.

The interaction between the VAPs and their partners occurs through the binding of the MSP domain within VAP to an FFAT (two phenalalanines in an acidic tract) motif located on the binding partner. The consensus sequence for FFAT motifs is EFFDAxE, though this sequence can tolerate a high degree of variability [63]. A negative upstream flanker region aids in the interaction between VAP and the FFAT motif although serine phosphorylation can replace acidic residues in this region, thus allowing activation by phosphorylation [64–66]. While Kv2 channels do not possess a stereotypical FFAT sequence, we identified a noncanonical FFAT motif located within the PRC domain of both Kv2 channels using a series of protein chimeras. In Kv2.1 this motif is SFISCAT while in Kv2.2 it is SFTSCAT. Phosphorylated serine residues both within and upstream of this motif may generate the negative charge necessary for VAP interaction, which could explain the Kv2 phosphorylation requirement to form ER/PM junctions. This Kv2 FFAT motif-VAP MSP domain interaction at the ER/PM interface is illustrated in Figure 2. Note that the VAP-dependent Kv2.1 clustering also generates a domain potentially involved in cell-to-cell communication since the Kv2.1 beta subunit, AMIGO, is an adhesion molecule [67–69]. The bottom row of Figure 1 illustrates the effect of glutamate-induced Kv2.1 dephosphosphorylation that frees the channel C-terminus from the ER VAPs, thus resulting in Kv2.1 declustering, VAP dispersal, and cortical ER remodeling.

Based on discussions with our laboratory, the Trimmer group was able to independently confirm the Kv2-VAP interaction. Importantly, the Trimmer lab generated anti-VAP antibodies and used immuno-staining to confirm that endogenous VAPs were concentrated at Kv2 clusters in both rat brain and cultured rat hippocampal neurons [70]. This concentration of endogenous VAPs at native Kv2.1 clusters complements our redistribution experiments in HEK293 cells where Kv2 induction of ER/PM contacts concentrated ER VAPs at this microdomain [48]. Our FRET data, which suggests VAPs may homo-oligomerize within Kv2.1-induced ER/PM junctions independently of Kv2.1 binding, provides a possible mechanism for this robust redistribution. In addition, VAP homo-oligomers could simultaneously bind Kv2 channels to form junctions and bind other interactors, thus concentrating additional proteins to these membrane contact sites (see Figure 2). VAPs have a large interactome, including kinases, vesicle trafficking proteins, kinesins, AKAPs and lipid transfer proteins [65,71], all of which may play important roles at Kv2-induced ER/PM junctions.

## Current model for Kv2.1-induced ER/PM contact sites

**Figure 3.**
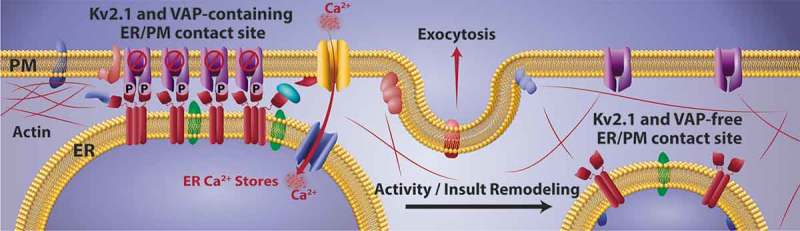
Model of the Kv2.1-induced ER/PM junction. Calcineurin-dependent dephosphorylation of the Kv2.1 C-terminus results in release from the ER VAPs, Kv2.1 declustering, loss of the ER/PM junction, VAP redistribution, and cortical ER retraction. The clustered Kv2.1 channels are proposed to be non-conducting under resting conditions [37]. Both the Kv2.1-containing and Kv2.1-free ER/PM junctions are trafficking hubs where dense core vesicle exocytosis [14,15] and membrane protein delivery is concentrated [44], perhaps in part due to these membrane contact sites residing in, or forming, “holes” in the cortical actin [36]. However, Kv2.1-containing membrane junctions are proposed to also concentrate SNARE proteins, for Kv2.1 contains VAMP-2, SNAP25 and syntaxin binding domains [72–74].

The model illustrated in Figure 3 agrees with data obtained in transfected HEK293 cells, cultured hippocampal neurons, rat brain slices and intact rat brain [48,70]. Here serine phosphorylation adjacent to the Kv2.1 C-terminal FFAT motif allows channel binding to VAPs present on the cytoplasmic surface of ER. This binding not only tethers the ER membrane to within 10 nm of the PM but it also concentrates VAPs to this microdomain and generates the Kv2 clustered phenotype. Given that VAP homo-oligomerization may occur at these sites to form a VAP scaffold, Kv2 channels effectively generate a site where additional VAP interactors can concentrate. This could provide these sites with the capability to perform further functions, from lipid transfer between ER and PM, to aiding ER calcium store refilling.

## Kv2-based regulation of ER/PM junctions and sensitivity to cellular insult

Individual Kv2.1 channels are capable of switching between being freely diffusive or clustered under homeostatic conditions [36], suggesting constant dynamic control over these microdomains that can be pushed either towards enlarging microdomains or their disassembly. Given the correct stimuli, Kv2 dephosphorylation breaks the Kv2-VAP link. The ER then retracts into the cytoplasm and VAPs redistribute throughout the ER. It is likely that the functions specifically associated with the Kv2-induced ER/PM contacts are then lost. Inversely, other stimuli can lead to the formation of Kv2-induced ER/PM contacts or enlargement of preexisting junctions, thus creating conditions under which functions can now be performed or performed to a greater degree.

There are several known conditions under which Kv2-induced ER/PM junctions are impacted, some of which this review has already covered. Recent electron microscopy work in cultured hippocampal neurons performed by Dr. Tao-Cheng [75,76] found a significant decrease in both size and number of ER/PM junctions after depolarization with high K^+^ or NMDA treatment. These findings fit neatly with the known effect neuronal activity has on Kv2.1 channel localization [31]. While mass dephosphorylation of Kv2.1 can both cause declustering as well as a hyperpolarizing shift in voltage dependence, we now know that these two events are not inextricably linked as once believed [37]. For instance, the cell cycle dependent ebb and flow of clustering behavior in COS-1 cells is not associated with changes in channel electrical activity [41]. Also, while Kv2.1 clusters predominantly contain non-conducting channels, the regulation of the non-conducting state occurs independently of channel localization [37,40]. Clearly, more work will need to be accomplished on the non-conducting state.

Kv2-induced ER/PM junctions are also impacted by cellular insult and disease. As previously mentioned, Kv2.1 clusters disperse in response to hypoxic or ischemic conditions [32,33]. Kv2.2 appears to be less sensitive to this insult-induced declustering [7] and this sensitivity or resistance to ER/PM junction rearrangement may be one reason cells have opted to use one channel over the other. Kv2.1 clusters are enlarged in some rat models of Alzheimer’s disease with a coupled decrease in K^+^ current density that may be due to increased oxidative stress during development [77]. Human mutations predicted to disrupt Kv2.1-VAP interaction without impacting channel electrical activity are associated with developmental delay [78]. There is also a great body of work performed by the Fyffe lab examining Kv2.1 in rat motoneurons and C-bouton synapses [9,79–81]. In 2014 this group discovered that Kv2.1 clusters dispersed in response to peripheral nerve injury in both ipsilateral and contralateral limbs [81]. Curiously, without reinnervation Kv2.1 clusters reformed over time, but with reinnervation they did not.

Determining the functions associated with Kv2-induced ER/PM junctions and how this microdomain can be targeted to enhance neuroprotection is an area of new importance. Recently, work by the Aizenman and Shah labs revealed that Kv2.1-induced ER/PM junctions are necessary for apoptosis [82]. Interfering with the formation of Kv2.1-induced ER/PM junctions, without affecting channel electrical activity, disrupted the apoptotic stimulus-induced trafficking of Kv2.1 to the surface, thus interfering with the increase in K^+^ efflux necessary for apoptosis [19,20]. This inhibition of Kv2.1 delivery improved neuroprotection during oxidative stress [82]. These results fit well with our earlier work demonstrating that Kv2.1 ER/PM junctions serve as trafficking hubs for the delivery of Kv2.1 and other ion channels [44]. Future studies on this topic are certainly warranted.

## ER/PM junction composition and function in the intact brain

ER/PM junctions were first described in muscle cells by Porter & Palade in 1957 [83]. Their existence in neurons would be described just five years later [84]. Recent focused ion beam-scanning electron microscopy (FIB-SEM) based reconstructions of ER/PM junctions in rodent brain by the De Camilli group, indicate these membrane contact sites represent up to 12% of the neuronal soma [85]. These membrane contacts also exist in dendrites along the dendritic shaft and in axons within the varicosities which are the predominant sites for neurotransmitter release. Whether Kv2.1 is involved in these non-somatic contacts remains an open question.

In the intact brain these membrane junctions are predicted to contain multiple components at any one time, (e.g. Kv2 channels, junctophilins, STIM proteins, voltage-gated Ca^2+^ channels, Trp channels, extended synaptotagmins, etc.), and the specific composition of ER/PM junctions will depend on cell type. Junction composition will also be affected by cellular state, for example, the STIM-Orai complex will reside in junctions during periods of ER calcium store depletion, but not during other times, and we now know that Kv2-VAP complexes are regulated by neuronal activity and cellular insult in a phosphorylation-dependent manner [41,47,48,70,86–88]. In addition, ER/PM junctions within different compartments of a single cell may display different properties. For example Kv2.1-induced junctions on the AIS are more resistant to CO_2_- and kainate-induced declustering than junctions on the soma [89].

Accumulating evidence suggests ER/PM junctions are not homogeneous, but that within these microdomains there exists nanodomain heterogeneity. For example, different proteins capable of forming ER/PM junctions require different spacing between the PM and ER membrane. This requirement, as has been demonstrated by the Balla group, can cause exclusion of certain proteins from specific contact site regions based on size [91]. It is possible that within intact brain tissue a singular ER/PM junction is subdivided into various nanodomains which perform different functions. This may account for the “donut-like” localization pattern of some ER/PM junction resident proteins; the proteins which require less distance between membranes are forced to the center of the junction and those that require more distance between membranes are forced to the periphery [91]. Endogenous Kv2.1 clusters in neurons are sometimes reported to have such a “donut-like” pattern with proteins such as neuregulin-2 occupying the center space [91].

Since multiple proteins may be contributing to ER/PM junction structure in the intact brain, events which cause Kv2-VAP unbinding may not disrupt the junction, but rather may only lead to a loss of Kv2, VAP, and potential VAP interactors from this location, thus providing perhaps a more nuanced response to certain stimuli. The interplay between these various nanodomains and the inherent dynamics within the entire junction is an obvious area for future investigation as is the functionality provided by the Kv2-VAP interaction and how this functionality is disturbed during cellular insult.

## Future directions

Now that we have begun to understand the molecular composition of Kv2-induced ER/PM junctions, future studies can confront the question so far largely missing from the history reviewed above: why use a non-conducting ion channel to form ER/PM junctions in the first place, i.e. what functionality does the cell gain from this arrangement over any other protein? Perhaps the voltage-sensing S4 domains of Kv2.1 communicate membrane potential to downstream effectors as occurs at the skeletal muscle PM/sarcoplasmic reticulum junction. Another potential advantage of using a K^+^ channel to form ER/PM contacts relates to ER Ca^2+^ store homeostasis. Perhaps the clustered Kv2 channels become conducting during ER Ca^2+^ store depletion, thus providing a localized K^+^ counter current to enhance any localized Ca^2+^ influx. More work is necessary to determine the exact role of the Kv2.1 syntaxin binding domain in exocytosis, especially considering recent findings suggesting that this domain regulates dense core vesicle exocytosis at Kv2.1 clusters and can rescue insulin release in diabetic pancreatic beta cells [14,15]. The Kv2.1 beta subunit, AMIGO, which is a cell adhesion molecule [68], could serve to position ER/PM junctions adjacent to cell-cell contact sites. Indeed, intercellular AMIGO interactions could be responsible for the symmetrical ER/PM contacts observed by the De Camilli group at some somatic contact sites [85]. And why would such activity- and insult-sensitive structures exist if not for some form of intercellular communication? As this story of Kv2.1 clustering continues to move forward we anticipate both the resolution of these questions and new surprises.

## References

[1] Singer SJ, Nicolson GL. The fluid mosaic model of the structure of cell membranes. Science. 1972;175(4023):720–731. 4333397 10.1126/science.175.4023.720

[2] Frech GC, VanDongen AM, Schuster G, et al A novel potassium channel with delayed rectifier properties isolated from rat brain by expression cloning. Nature. 1989;340(6235):642–645. 2770868 10.1038/340642a0

[3] Hwang PM, Glatt CE, Bredt DS, et al A novel K+ channel with unique localizations in mammalian brain - molecular-cloning and characterization. Neuron. 1992;8(3):473–481. 1550672 10.1016/0896-6273(92)90275-i

[4] Hwang PMCA, Peng YW, Snyder SH CDRK and DRK1 K+ channels have contrasting localizations in sensory systems. Neuroscience. 1993;55(3):613–620. 8413924 10.1016/0306-4522(93)90427-h

[5] Hwang PM, Fotuhi M, Bredt DS, et al Contrasting immunohistochemical localizations in rat brain of two novel K+ channels of the Shab subfamily. J Neurosci. 1993;13(4):1569–1576. 8463836 10.1523/JNEUROSCI.13-04-01569.1993 PMC6576723

[6] Kihira Y, Hermanstyne TO, Misonou H Formation of heteromeric Kv2 channels in mammalian brain neurons. J Biol Chem. 2010;285(20):15048–15055. 20202934 10.1074/jbc.M109.074260 PMC2865335

[7] Bishop HI, Guan D, Bocksteins E, et al Distinct cell- and layer-specific expression patterns and independent regulation of Kv2 channel subtypes in cortical pyramidal neurons. J Neurosci. 2015;35(44):14922–14942. 26538660 10.1523/JNEUROSCI.1897-15.2015 PMC4635138

[8] Du J, Tao-Cheng JH, Zerfas P, et al The K+ channel, Kv2.1, is apposed to astrocytic processes and is associated with inhibitory postsynaptic membranes in hippocampal and cortical principal neurons and inhibitory interneurons. Neuroscience. 1998;84(1):37–48. 9522360 10.1016/s0306-4522(97)00519-8

[9] Muennich EAL, Fyffe REW Focal aggregation of voltage-gated, Kv2.1 subunit-containing, potassium channels at synaptic sites in rat spinal motoneurones. J Physiol-London. 2004;554(3):673–685. 14608003 10.1113/jphysiol.2003.056192 PMC1664801

[10] Yazulla S, Studholme KM Differential distribution of Shaker-like and Shab-like K+-channel subunits in goldfish retina and retinal bipolar cells. J Comp Neurol. 1998;396(1):131–140. 9623892 10.1002/(sici)1096-9861(19980622)396:1<131::aid-cne10>3.0.co;2-s

[11] O’Connell KM, Whitesell JD, Tamkun MM Localization and mobility of the delayed-rectifer K+ channel Kv2.1 in adult cardiomyocytes. Am J Physiol Heart Circ Physiol. 2008;294(1):H229–37. 17965280 10.1152/ajpheart.01038.2007

[12] Amberg GC, Santana LF Kv2 channels oppose myogenis constriction of rat cerebral arteries. Am J Physiol Cell Physiol. 2006;291(2):C348–C56. 10.1152/ajpcell.00086.2006 16571867

[13] Epperson A, Bonner HP, Ward SM, et al Molecular diversity of Kvα- and β-subunit expression in canine gastrointestinal smooth muscles. Neuroregulation Motil. 1999;277(1):G127–G36. 10.1152/ajpgi.1999.277.1.G127 10409159

[14] Fu J, Dai X, Plummer G, et al Kv2.1 clustering contributes to insulin exocytosis and rescues human beta-cell dysfunction. Diabetes. 2017;66(7):1890–1900. 28607108 10.2337/db16-1170 PMC5482075

[15] Greitzer-Antes D, Xie L, Qin T, et al Kv2.1 clusters on beta-cell plasma membrane act as reservoirs that replenish pools of newcomer insulin granule through their interaction with syntaxin-3. J Biol Chem. 2018;16(002703):002703. 10.1074/jbc.RA118.002703 PMC5936832 29549124

[16] Du J, Haak LL, Phillips-Tansey E, et al Frequency-dependent regulation of rat hippocampal somato-dendritic excitability by the K+ channel subunit Kv2.1. J Physiol. 2004;522(1):19–31. 10.1111/j.1469-7793.2000.t01-2-00019.xm PMC2269745 10618149

[17] Xu H, Barry DM, Li H, et al Attenuation of the slow component of delayed rectification, action potential prolongation, and triggered activity in mice expressing a dominant-negative Kv2 alpha subunit. Circ Res. 1999;85(7):623–633. 10506487 10.1161/01.res.85.7.623

[18] Jacobson DA, Kuznetsov A, Lopez JP, et al Kv2.1 ablation alters glucose-induced islet electrical activity, enhancing insulin secretion. Cell Metab. 2007;6(3):229–235. 17767909 10.1016/j.cmet.2007.07.010 PMC2699758

[19] Pal S, Hartnett KA, Nerbonne JM, et al Mediation of neuronal apoptosis by Kv2.1-encoded potassium channels. J Neurosci. 2003;23(12):4798–4802. 12832499 10.1523/JNEUROSCI.23-12-04798.2003 PMC2945225

[20] Pal SK, Takimoto K, Aizenman E, et al Apoptotic surface delivery of K+ channels. Cell Death Differ. 2006;13(4):661–667. 16273079 10.1038/sj.cdd.4401792 PMC1403161

[21] Redman PT, Hartnett KA, Aras MA, et al Regulation of apoptotic potassium currents by coordinated zinc-dependent signalling. J Physiol. 2009;587(Pt 18):4393–4404. 19622611 10.1113/jphysiol.2009.176321 PMC2766646

[22] Redman PT, He K, Hartnett KA, et al Apoptotic surge of potassium currents is mediated by p38 phosphorylation of Kv2.1. Proc Natl Acad Sci U S A. 2007;104(9):3568–3573. 17360683 10.1073/pnas.0610159104 PMC1805571

[23] Thiffault I, Speca DJ, Austin DC, et al A novel epileptic encephalopathy mutation in KCNB1 disrupts Kv2.1 ion selectivity, expression, and localization. J Gen Physiol. 2015;146(5):399–410. 26503721 10.1085/jgp.201511444 PMC4621747

[24] Torkamani A, Bersell K, Jorge BS, et al De novo KCNB1 mutations in epileptic encephalopathy. Ann Neurol. 2014;76(4):529–540. 25164438 10.1002/ana.24263 PMC4192091

[25] Speca DJ, Ogata G, Mandikian D, et al Deletion of the Kv2.1 delayed rectifier potassium channel leads to neuronal and behavioral hyperexicitability. Genes Brain Behav. 2014;13:394–408. 24494598 10.1111/gbb.12120 PMC4077602

[26] Trimmer JS Immunological identification and characterization of a delayed rectifier K+ channel polypeptide in rat brain. Proc Natl Acad Sci U S A. 1991;88(23):10764–10768. 1961744 10.1073/pnas.88.23.10764 PMC53011

[27] Scannevin RH, Murakoshi H, Rhodes KJ, et al Identification of a cytoplasmic domain important in the polarized expression and clustering of the Kv2.1 K+ channel. J Cell Biol. 1996;135(6 Pt 1):1619–1632. 8978827 10.1083/jcb.135.6.1619 PMC2133974

[28] Shi G, Kleinklaus AK, Marrion NV, et al Properties of Kv2.1 K+ channels expressed in transfected mammalian cells. J Biol Chem. 1994;269(37):23204–23211. 8083226

[29] Lim ST, Antonucci DE, Scannevin RH, et al A novel targeting signal for proximal clustering of the Kv2.1 K+ channel in hippocampal neurons. Neuron. 2000;25(2):385–397. 10719893 10.1016/s0896-6273(00)80902-2

[30] Antonucci DE, Lim ST, Vassanelli S, et al Dynamic localization and clustering of dendritic Kv2.1 voltage-dependent potassium channels in developing hippocampal neurons. Neuroscience. 2001;108(1):69–81. 10.1016/s0306-4522(01)00476-6 11738132

[31] Misonou H, Mohapatra DP, Park EW, et al Regulation of ion channel localization and phosphorylation by neuronal activity. Nat Neurosci. 2004;7(7):711–718. 15195093 10.1038/nn1260

[32] Misonou H, Mohapatra DP, Menegola M, et al Calcium- and metabolic state-dependent modulation of the voltage-dependent Kv2.1 channel regulates neuronal excitability in response to ischemia. J Neurosci. 2005;25(48):11184–11193. 16319318 10.1523/JNEUROSCI.3370-05.2005 PMC6725654

[33] Misonou H, Mohapatra DP, Trimmer JS Kv2.1: a voltage-gated k+ channel critical to dynamic control of neuronal excitability. Neurotoxicology. 2005;26(5):743–752. 15950285 10.1016/j.neuro.2005.02.003

[34] Surmeier DJ, Foehring R A mechanism for homeostatic plasticity. Nat Neurosci. 2004;7(7):691–692. 15220926 10.1038/nn0704-691

[35] O’Connell KM, Rolig AS, Whitesell JD, et al Kv2.1 potassium channels are retained within dynamic cell surface microdomains that are defined by a perimeter fence. J Neurosci. 2006;26(38):9609–9618. 16988031 10.1523/JNEUROSCI.1825-06.2006 PMC6674455

[36] Tamkun MM, O’Connell KM, Rolig AS A cytoskeletal-based perimeter fence selectively corrals a sub-population of cell surface Kv2.1 channels. J Cell Sci. 2007;120(Pt14):2413–2423. 17606996 10.1242/jcs.007351

[37] O’Connell KM, Loftus R, Tamkun MM Localizationdependent activity of the Kv2.1 delayed-rectifier K+ channel. Proc Natl Acad Sci U S A. 2010;107(27):12351–12356. 10.1073/pnas.1003028107 PMC2901471 20566856

[38] Benndorf K, Koopmann R, Lorra C, Pongs O Gating and conductance properties of a human delayed rectifier K+ channel expressed in frog oocytes. J Physiol. 1994;477(Pt 1):1–14. 10.1113/jphysiol.1994.sp020166 PMC1155569 8071876

[39] Baver SB, O’Connell KM The C-terminus of neuronal Kv2.1 channels is required for channel localization and targeting but not for NMDA-receptor-mediated regulation of channel function. Neuroscience. 2012;217:56–66. 22554782 10.1016/j.neuroscience.2012.04.054 PMC3383376

[40] Fox PD, Loftus RJ, Tamkun MM Regulation of Kv2.1 K(+) conductance by cell surface channel density. J Neurosci. 2013;33(3):1259–1270. 23325261 10.1523/JNEUROSCI.3008-12.2013 PMC3711267

[41] Cobb MM, Austin DC, Sack JT, et al Cell cycle-dependent changes in localization and phosphorylation of the plasma membrane Kv2.1 K+ channel impact endoplasmic reticulum membrane contact sites in COS-1 cells. J Biol Chem. 2015;290(49):29189–29201. 26442584 10.1074/jbc.M115.690198 PMC4705925

[42] Feinshreiber L, Singer-Lahat D, Friedrich R, et al Non-conducting function of the Kv2.1 channel enables it to recruit vesicles for release in neuroendocrine and nerve cells. J Cell Sci. 2010;123(Pt11):1940–1947. 20484665 10.1242/jcs.063719

[43] Singer-Lahat D, Sheinin A, Chikvashvili D, et al K+ channel facilitation of exocytosis by dynamic interaction with syntaxin. J Neurosci. 2007;27(7):1651–1658. 17301173 10.1523/JNEUROSCI.4006-06.2007 PMC6673747

[44] Deutsch E, Weigel AV, Akin EJ, et al Kv2.1 cell surface clusters are insertion platforms for ion channel delivery to the plasma membrane. Mol Biol Cell. 2012;23(15):2917–2929. 22648171 10.1091/mbc.E12-01-0047 PMC3408418

[45] Fox PD, Haberkorn CJ, Weigel AV, et al Plasma membrane domains enriched in cortical endoplasmic reticulum function as membrane protein trafficking hubs. Mol Biol Cell. 2013;24(17):2703–2713. 23864710 10.1091/mbc.E12-12-0895 PMC3756922

[46] Tanabe T, Mikami A, Numa S, et al Cardiac-type excitation-contraction coupling in dysgenic skeletal muscle injected with cardiac dihydropyridine receptor cDNA. Nature. 1990;344(6265):451–453. 2157159 10.1038/344451a0

[47] Fox PD, Haberkorn CJ, Akin EJ, et al Induction of stable ER-plasma-membrane junctions by Kv2.1 potassium channels. J Cell Sci. 2015;128(11):2096–2105. 25908859 10.1242/jcs.166009 PMC4457025

[48] Johnson B, Leek AN, Sole L, et al Kv2 potassium channels form endoplasmic reticulum/plasma membrane junctions via interaction with VAPA and VAPB. Proc Natl Acad Sci U S A. 2018;115(31):E7331–E40. 29941597 10.1073/pnas.1805757115 PMC6077746

[49] Kirmiz M, Palacio S, Thapa P, et al Remodeling neuronal ER-PM junctions is a conserved nonconducting function of Kv2 plasma membrane ion channels. Mol Biol Cell. 2018;29(20):2410–2432. 30091655 10.1091/mbc.E18-05-0337 PMC6233057

[50] Skehel PA, Martin KC, Kandel ER, et al A VAMP-binding protein from Aplysia required for neurotransmitter release. Science. 1995;269(5230):1580–1583. 7667638 10.1126/science.7667638

[51] Nishimura Y, Hayashi M, Inada H, et al Molecular cloning and characterization of mammalian homologues of vesicle-associated membrane protein-associated (VAMP-associated) proteins. Biochem Biophys Res Commun. 1999;254(1):21–26. 9920726 10.1006/bbrc.1998.9876

[52] Loewen CJR, Levine TP A highly conserved binding site in Vesicle-associated membrane protein-Associated Protein (VAP) for the FFAT motif of lipid-binding proteins. J Biol Chem. 2005;280(14):14097–14104. 15668246 10.1074/jbc.M500147200

[53] Kaiser SE, Brickner JH, Reilein AR, et al Structural basis of FFAT motif-mediated ER targeting. Structure. 2005;13(7):1035–1045. 16004875 10.1016/j.str.2005.04.010

[54] Lev S, Ben Halevy D, Peretti D, et al The VAP protein family: from cellular functions to motor neuron disease. Trends Cell Biol. 2008;18(6):282–290. 18468439 10.1016/j.tcb.2008.03.006

[55] Amarilio R, Ramachandran S, Sabanay H, Lev S Differential regulation of endoplasmic reticulum structure through VAP-Nir protein interaction. J Biol Chem. 2005;280(7):5934–5944. 15545272 10.1074/jbc.M409566200

[56] Nishimura AL, Mitne-Neto M, Silva HCA, Richieri-Costa A, et al A mutation in the vesical-trafficking protein VAPB causes late-onset spinal muscular atrophy and amyotrophic lateral sclerosis. Am J Hum Genet. 2004;75(5):822–831. 15372378 10.1086/425287 PMC1182111

[57] Teuling E, Ahmed S, Haasdijk E, et al Motor neuron disease-associated mutant vesicle-associated membrane protein-associated protein (VAP) B recruits wild-type VAPs into endoplasmic reticulum-derived tubular aggregates. J Neurosci. 2007;27(36):9801–9815. 17804640 10.1523/JNEUROSCI.2661-07.2007 PMC6672975

[58] Mitne-Neto M, Machado-Costa M, Marchetto MC, et al Downregulation of VAPB expression in motor neurons derived from induced pluripotent stem cells of ALS8 patients. Hum Mol Genet. 2011;20(18):3642–3652. 21685205 10.1093/hmg/ddr284 PMC3159551

[59] Pennetta G, Hiesinger PR, Fabian-Fine R, Meinertzhagen IA, et al Drosophila VAP-33A directs bouton formation at neuromuscular junction in a dosage-dependent manner. Neuron. 2002;25(2):291–306. 10.1016/s0896-6273(02)00769-9 12160747

[60] Appel N, Schuller T, Penin F, Bartenschlager R From structure to function: new insights into hepatitis C virus RNA replication. J Biol Chem. 2006;281:9833–9836. 16407182 10.1074/jbc.R500026200

[61] Barajas D, Xu K, de Castro Martin IF, et al Co-opted oxysterol-binding ORP and VAP proteins channel sterols to RNA virus replication sites via membrane contact sites. PLoS Pathog. 2014;10(10):e1004388. 25329172 10.1371/journal.ppat.1004388 PMC4199759

[62] McCune BT, Tang W, Lu J, et al Noroviruses co-opt the function of host proteins VAPA and VAPB for replication via a phenylalanine-phenylalanine-acidic-tract-motif mimic in nonstructural viral protein NS1/2. MBio. 2017;8(4):e00668–17. 28698274 10.1128/mBio.00668-17 PMC5513711

[63] Mikitova V, Levine TP Analysis of the key elements of FFAT-like motifs identifies new proteins that potentially bind VAP on the ER, including two AKAPs and FAPP2. Plos One. 2012;7(1):e30455. 22276202 10.1371/journal.pone.0030455 PMC3261905

[64] Kumagai K, Kawano-Kawada M, Hanada K Phosphorylation of the cerimide transport protein CERT at serine 315 in the interaction with VAMP-associated protein (VAP) for inter-organelle trafficking of ceramide in mammalian cells. J Biol Chem. 2014;289(15):10748–10760. 24569996 10.1074/jbc.M113.528380 PMC4036191

[65] Murphy SE, Levine TP VAP, a versatile access point for the endoplasmic reticulum: review and analysis of FFAT-like motifs in the VAPome. Biochim Biophys Acta. 2016;1861(8 Pt B):952–961. 26898182 10.1016/j.bbalip.2016.02.009

[66] Weber-Boyvat M, Kentala H, Lilja J, et al OSBP-related protein 3 (ORP3) coupling with VAMP-associated protein A regulates R-Ras activity. Exp Cell Res. 2015;331(2):278–291. 25447204 10.1016/j.yexcr.2014.10.019

[67] Bishop HI, Cobb MM, Kirmiz M, et al Kv2 ion channels determine the expression and localization of the associated AMIGO-1 cell adhesion molecule in adult brain neurons. Front Mol Neurosci. 2018;11:1. 29403353 10.3389/fnmol.2018.00001 PMC5780429

[68] Kuja-Panula J, Kiiltomaki M, Yamashiro T, Rouhiainen A, et al AMIGO, a transmembrane protein impicated in axon tract development, defines a novel protein family with leucine-rich repeats. J Cell Biol. 2003;160:963–973. 12629050 10.1083/jcb.200209074 PMC2173769

[69] Peltola MA, Kuja-Panula J, Lauri SE, et al AMIGO is an auxiliary subunit of the Kv2.1 potassium channel. EMBO Rep. 2011;12(12):1293–1299. 22056818 10.1038/embor.2011.204 PMC3245694

[70] Kirmiz M, Vierra NC, Palacio S, et al Identification of VAPA and VAPB as Kv2 channel-interacting proteins defining endoplasmic reticulum-plasma membrane junctions in mammalian brain neurons. J Neurosci. 2018;38(35):7562–7584. 30012696 10.1523/JNEUROSCI.0893-18.2018 PMC6113906

[71] Huttlin EL, Ting L, Bruckner RJ, et al The BioPlex network: a systematic exploration of the human interactome. Cell. 2015;162(2):425–440. 26186194 10.1016/j.cell.2015.06.043 PMC4617211

[72] Leung YM, Kang Y, Gao X, et al Syntaxin 1A binds to the cytoplasmic C terminus of Kv2.1 to regulate channel gating and trafficking. J Biol Chem. 2003;278(19):17532–17538. 12621036 10.1074/jbc.M213088200

[73] Lvov A, Chikvashvili D, Michaelevski I, et al VAMP2 interacts directly with the N terminus of Kv2.1 to enhance channel inactivation. Pflugers Arch. 2008;456(6):1121–1136. 18542995 10.1007/s00424-008-0468-7

[74] MacDonald PE, Wang G, Tsuk S, et al Synaptosome-associated protein of 25 kilodaltons modulates Kv2.1 voltage-dependent K+ channels in neuroendocrine islet β-cells through an interaction with the channel N terminus. Mol Endocrinol. 2002;16(11):2452–2461. 12403834 10.1210/me.2002-0058

[75] Tao-Cheng J-H Activity-dependent decrease in contact areas between subsurface cisterns and plasma membrane of hippocampal neurons. Mol Brain. 2018;11:23. 29661253 10.1186/s13041-018-0366-7 PMC5902880

[76] Tao-Cheng J-H Stimulation-induced structural changes at the nucleus, endoplasmic reticulum and mitochondria of hippocampal neurons. Mol Brain. 2018;11:44. 30049284 10.1186/s13041-018-0387-2 PMC6062868

[77] Frazzini V, Guarnieri S, Bomba M, et al Altered Kv2.1 functioning promotes increased excitability in hippocampal neurons of an Alzheimer’s disease mouse model. Cell Death Dis. 2016;7(2):e2100. 10.1038/cddis.2016.18 PMC5399189 26890139

[78] de Kovel CGF, Syrbe S, Brilstra EH, et al Neurodevelopmental disorders caused by De Novo variants in KCNB1 genotypes and phenotypes. JAMA Neurol. 2017;74(10):1228–1236. 28806457 10.1001/jamaneurol.2017.1714 PMC5710242

[79] Deardorff AS, Romer SH, Sonner PM, et al Swimming against the tide: investigations of the C-bouton synapse. Front Neural Circuits. 2014;8:106. 25278842 10.3389/fncir.2014.00106 PMC4167003

[80] Romer SH, Deardorff AS, Fyffe RE Activity-dependent redistribution of Kv2.1 ion channels on rat spinal motoneurons. Physiol Rep. 2016;4(22):e13039. 27884958 10.14814/phy2.13039 PMC5358001

[81] Romer SH, Dominguez KM, Gelpi MW, et al Redistribution of Kv2.1 ion channels on spinal motoneurons following peripheral nerve injury. Brain Res. 2014;1547:1–15. 24355600 10.1016/j.brainres.2013.12.012 PMC3970712

[82] Justice JA, Shulien AJ, He K, et al Disruption of Kv2.1 somato-dendritic clusters prevents the apoptogenic increase of potassium currents. Neuroscience. 2017;354(23):158–167. 28461216 10.1016/j.neuroscience.2017.04.034 PMC5709998

[83] Porter KR, Palade GE Studies on the endoplasmic reticulum. J Cell Biol. 1957;3(2):269–300. 10.1083/jcb.3.2.269 PMC2224093 13438910

[84] Rosenbluth J. Subsurface cisterns and their relationship to the neuronal plasma membrane. J Cell Biol. 1962;13(3):405–421. 14493991 10.1083/jcb.13.3.405 PMC2106078

[85] Wu YM, Whiteus C, Xu CS, et al Contacts between the endoplasmic reticulum and other membranes in neurons. Proc Natl Acad Sci U S A. 2017;114(24):E4859–E67. 28559323 10.1073/pnas.1701078114 PMC5474793

[86] Huang GN, Zeng W, Kim JY, et al STIM1 carboxyl-terminus activates native SOC, I(crac) and TRPC1 channels. Nat Cell Biol. 2006;8(9):1003–1010. 16906149 10.1038/ncb1454

[87] Korzeniowski MK, Popovic MA, Szentpetery Z, et al Dependence of STIM1/Orai1-mediated calcium entry on plasma membrane phosphoinositides. J Biol Chem. 2009;284(31):21027–21035. 19483082 10.1074/jbc.M109.012252 PMC2742867

[88] Korzeniowski MK, Manjarres IM, Varnai P, et al Activation of STIM1-Orai1 involves an intramolecular switching mechanism. Sci Signal. 2010;3(148):ra82. 21081754 10.1126/scisignal.2001122 PMC3408607

[89] King AN, Manning CF, Trimmer JS A unique ion channel clustering domain on the axon initial segment of mammalian neurons. J Comp Neurol. 2014;522(11):2594–2608. 24477962 10.1002/cne.23551 PMC4133991

[90] Varnai P, Toth B, Toth DJ, et al Visualization and manipulation of plasma membrane-endoplasmic reticulum contact sites indicates the presence of additional molecular components within the STIM1-Orai1 complex. J Biol Chem. 2007;282(40):29678–29690. 17684017 10.1074/jbc.M704339200

[91] Vullhorst D, Mitchell RM, Keating C, et al A negative feedback loop controls NMDA receptor function in cortical interneurons via neuregulin 2/ErbB4 signalling. Nat Commun. 2015;6:7222. 26027736 10.1038/ncomms8222 PMC4451617

